# Does seasonality affect snoring? A study based on international data from the past decade

**DOI:** 10.1007/s11325-022-02717-9

**Published:** 2022-10-11

**Authors:** Ping Wang, Cai Chen, Xingwei Wang, Ningling Zhang, Danyang Lv, Wei Li, Fulai Peng, Xiuli Wang

**Affiliations:** 1 State Key Laboratory of Precision Measurement Technology and Instruments, Tianjin University, Tianjin, 300072 China; 2 Shandong Academy of Chinese Medicine, Jinan, 250014 China; 3 Shandong Institute of Advanced Technology Chinese Academy of Sciences, Jinan, 250000 China; 4 Biomedical Engineering Institute, School of Control Science and Engineering, Shandong University, Jinan, 250061 China; 5 Department of Pulmonary and Critical Care Medicine, Yantai Yeda Hospital, Yantai, China

**Keywords:** Snoring, Google Trends, Baidu Index, Seasonal changes

## Abstract

**Background:**

Though snoring is often regarded as a harmless condition that coincides with sound sleep, it is a sleep disorder that can be a potential indicator of more severe conditions such as sleep apnea syndrome. In the present study, we investigated the association between seasonal variations and snoring.

**Method:**

Search index for snoring (SIS) data were obtained from Google Trends and Baidu Index. SIS data were collected for the USA, India, Germany, Russia, Japan, Australia, China, and Brazil from 2011 to 2020, with the periodicity of the relationship between seasonal time series data and snoring evaluated using a time series decomposition model.

**Result:**

The highest average SIS growth rates from 2011 to 2020 were observed for Brazil, Japan, and Germany, with average SIS values of 94%, 68%, and 49%, respectively. The SIS of the USA, Russia, Japan, Brazil, Australia, Germany, and India increased by 22.3%, 12.4%, 11.9%, 35.4%, 12.3%, 28.0%, and 55.8%, respectively, in comparison with their SIS values in 2019, whereas for China, it decreased by 13.7%. Relative to countries in the southern hemisphere, those in the northern hemisphere showed comparable SIS trends, increasing from September to February and decreasing from March to August.

**Conclusion:**

The SIS data showed cyclical changes over the study period. The search index for snoring increased during the cold season or the heating season, suggesting that snoring is associated with seasonal changes.

**Supplementary Information:**

The online version contains supplementary material available at 10.1007/s11325-022-02717-9.

## Introduction

Snoring is caused by vibrations in the soft tissues of the upper airway because of breathing during sleep [1]. Snoring is broadly classified into two categories, namely physiological snoring and pathological snoring [2]. Physiological snoring is generally benign and observed in healthy individuals occasionally because of physical fatigue and improper sleep positioning [3], potentially occurring because of excessive pharyngeal muscle relaxation during the deep stage of sleep after alcohol consumption. Such snoring has a short-term effect on the human body and does not result in any noteworthy complications. In contrast, pathological snoring occurs because of insufficient air intake and excessive airflow resistance in breathing, thus affecting air exchange. Pathological snoring is among the most commonly observed clinical manifestations of obstructive sleep apnea–hypopnea syndrome [4].

Several conditions can lead to snoring. Obesity causes snoring as obese people have more fat than non-obese people, and their muscles relax when they sleep, causing airway stenosis or obstruction [5]. Tonsil, soft palate, and tongue body hypertrophies, long uvula, throat relaxation, and tongue suffix also contribute to snoring. Local obesity or the deformation of these organs and tissues may cause pharyngeal cavity stenosis and poor respiratory airflow [6, 7]. In addition, nasal septum deviation and nasal polyps can cause nasal stenosis, resulting in airflow resistance, thus causing snoring. Maxillofacial deformities, such as micrognathia, can cause airflow obstruction during nasopharyngeal breathing [8, 9]. Sleeping positions and fatigue can lead to snoring.

In a survey of 12,643 Hungarians, 50% self-reported habitual snoring [10], 37% of males reported loud snoring with breathing pauses, whereas 23% were habitual snorers. In contrast, Sogut et al. [11] reported a 4% prevalence of habitual snoring among adolescents from Manisa Province. These individuals reported higher rates of other nocturnal symptoms including difficulty breathing, restless sleep, and mouth breathing, and observed apneas relative to individuals that did not report snoring. In another study from Pakistan, out of 2497 individuals, 32% self-reported snoring, and 25% experienced a combination of sleep-disordered breathing symptoms [12]. In Japan, habitual snoring affects 24% of males and 10% of females [13], whereas prevalence rates in the USA and Europe range from 6.8 to 41.9% [14, 15].

Snoring is generally considered a harmless sound associated with deep sleep. However, snoring can indicate obstructive sleep apnea and related comorbidities. Snoring can contribute to the development of several conditions including diabetes, hypertension, cardiovascular diseases, cerebrovascular diseases, and coronary heart diseases. A study showed that snoring was related to a 46% higher risk of stroke incidence [16]. In a report, the pooled odds ratios (ORs) corresponding to the relationship between snoring, metabolic syndrome, and its components including hypertension, hyperglycemia, low high-density lipoprotein levels, high triglyceride levels, and abdominal obesity were 1.61, 1.23, 1.05, 1.09, 1.08, and 1.75, respectively [17]. Another perspective journaling-based study showed that approximately 60% of snorers and their bed partners reported morning headaches at least once within 90 days [18]. A cross-sectional study revealed that compared with non-snorers, the ORs of hypertension were 1.17% (95% confidence interval [CI]: 1.12–1.23) for frequent snorers and 1.12 (95% CI: 1.07–1.18) for occasional snorers [19]. A meta-analysis showed that compared with non-snorers, an increased risk of hypertension (OR: 1.32, 95% CI: 1.23–1.42) [20] was observed in snorers. Simple snoring was significantly associated with increased odds of hypertension (OR: 1.730, 95% CI: 1.130–2.650) and abdominal obesity (OR: 1.810, 95% CI: 1.063–3.083) among all participants. The physiological mechanisms underlying the potential cardiovascular effects of snoring are partly explained by oxidative stress, increased inflammatory responses, and prolonged sympathetic activation because of partial or complete upper airway obstruction [20]. Studies have shown that snoring may reduce sleep quality, and poor sleep has been linked to high blood pressure and may even increase the risk of developing it.

According to a study involving 72,885 Koreans, those who snore 6 + /week were associated with increased odds for metabolic syndrome with OR of 2.07 (95% CI: 1.91–2.25) for men and 1.45 (95% CI: 1.33–1.58) for women [21]. In a study involving 3948 participants, those with heavy snoring had a 1.84-fold higher risk of prediabetes (95% CI: 1.09–2.29) and a 2.24-fold higher risk of diabetes (95% CI: 1.84–2.95) [22]. Intermittent hypoxia and hypercapnia due to snoring induce upper airway obstruction, stimulate the sympathetic and hypothalamic–pituitary–adrenal axes, and increase catecholamine and cortisol levels, respectively, causing glucose intolerance and insulin resistance, thus leading to type 2 diabetes mellitus [23]. Hypoxia also increases levels of counter-regulatory hormones and activates proinflammatory cytokines, which may mediate peripheral insulin resistance and induce diabetes [24].

Given the high prevalence of snoring and its association with potentially severe complications, factors contributing to pathological snoring need to be investigated. Snoring is a multifactorial breathing disorder, with abnormalities in the upper airway structure and obesity as the two most common causes [25]. Abnormalities in the upper airway structure include tongue base, tonsil, and turbinate hypertrophies; jaw retraction; soft palate length; and nasal septum deviation [6, 7]. Obese individuals possess greater volumes of neck tissues than non-obese individuals, resulting in increased pressure on the respiratory tract that can disrupt airflow and thus cause snoring.

Snoring has also been linked to environmental factors. A cross-sectional study involving 5204 adults showed that an increase in relative humidity was associated with a decrease in the snoring index [26]. Habitual snoring (≥ 3 nights/week) was associated with air pollutant concentrations. The prevalence of habitual snoring among children in the southern and central neighborhoods of Tehran, Iran, where pollution was higher, was 24.5% and 12.1%, respectively, compared with 7.0% and 7.7% in the northern and eastern neighborhoods [27]. People exposed to high concentration of nitrogen dioxide (OR = 1.037, 95% CI 1.012–1.063) and low temperature (OR = 0.866, 95% CI 0.777–0.967) were more likely to snore [28]. These studies showed the effects of air pollution, temperature, and humidity on snoring, all of which were affected by seasonal changes. As seasonality impacts humidity, air pressure, and ambient temperature, we hypothesized a potential relationship between seasonal changes and snoring.

To date, investigating the relationship between seasonal changes and snoring worldwide has been challenging. However, the advent of increasingly robust internet tools has made such analyses possible, with both Google Trends and Baidu Index having previously been used to survey the prevalence of related diseases [29, 30]. Therefore, we used these online data sources in this study to explore the relationship between seasonal changes and snoring.

## Methods

### Data sources

This study was designed to explore the relationship between seasonal changes and snoring. Although collecting the global data on snoring is not feasible, Internet-derived search data can serve as a valuable proxy for the mapping of real-world phenomena, reflecting, for example, the epidemiology of a given disease [31]. Indeed, such data have been successfully applied to detect and analyze COVID-19 outbreaks in the recent past [32]. Accordingly, in the present study, snoring-related data from 2011 to 2020 were derived from Google Trends for the USA, India, Germany, Russia, Japan, Australia, and Brazil. As Google cannot be used for data search in China, snoring-related data from China during this period was obtained from the Baidu Index (https://index.baidu.com/v2/index.html#/).

We obtained the search index for snoring by searching for the term “snoring” in Google Trends and Baidu Index as the presence of the word “snoring” in a paragraph or sentence was picked up by Google Trends and Baidu. The search index was based on the web search volume of a certain area in a current time, taking the keywords as the statistical objects, and scientifically analyzing and calculating the weighted sum of the search frequency of each keyword in the web search. In other words, the search index is equal to the proportion of the total search volume of keyword searches in a current area at a particular time.

### Statistical analysis

The periodicity effects linking seasonal time series data and snoring were analyzed through a time series decomposition approach. Specifically, to explore the effects of seasonality and trends, these data were decomposed into seasonal and trend series [33]. For this purpose, the following model was adapted: Y t = T t + S t + E t, where Y t corresponds to the original times series data (search volume indexes for snoring), while T t, S t, and E t denote the trend component, the seasonal component (seasonal factor), and the residual component, respectively. Absolute cumulative and year-on-year increments were used to describe variations in the search index for snoring (SIS). A seasonal time series decomposition model was performed in the R software.

## Results

**Fig. 1:**
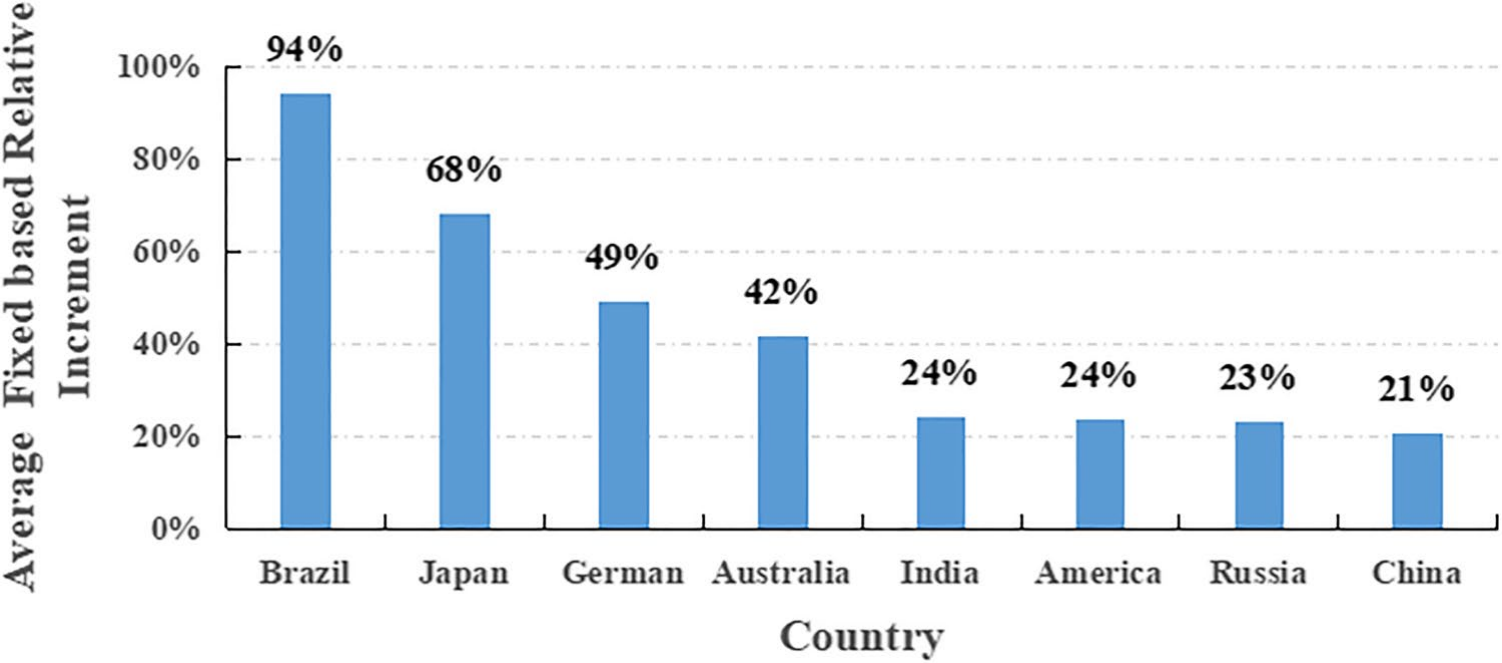
Average cumulative SIS changes from 2011 to 2020

Absolute cumulative changes in the search index for snoring (SIS) over the study period are compiled in Table 1. Compared to the SIS values in 2011, those for the USA, Russia, Japan, Brazil, Australia, Germany, India, and China in 2020 had increased by 64.5%, 42.9%, 112.4%, 262.9%, 76.8%, 111.3%, 85.6%, and 2.2%, respectively. Figure 1 provides the summary statistics for average cumulative changes in the SIS over the study period. The top 3 countries with respect to average SIS growth rates were Brazil, Japan, and Germany, with average SIS changes of 94%, 68%, and 49%, respectively.

**Table 1:** Absolute cumulative changes in the search index for snoring (SIS) from 2011 to 2020

Year	America	Russia	Japan	Brazil	Australia	German	India	China*
2011	-	-	-	-	-	-	-	-
2012	2.5%	12.2%	19.0%	− 2.2%	9.1%	11.7%	− 1.8%	36.3%
2013	3.0%	19.4%	32.2%	− 6.8%	5.5%	15.8%	9.0%	33.6%
2014	3.3%	36.7%	48.4%	− 2.9%	25.4%	18.4%	13.2%	20.9%
2015	16.9%	27.6%	61.4%	8.6%	44.3%	58.3%	34.1%	15.7%
2016	19.4%	13.8%	74.5%	43.9%	53.5%	45.5%	18.0%	19.7%
2017	33.5%	15.3%	80.8%	178.4%	55.3%	52.6%	21.6%	25.8%
2018	34.6%	15.3%	94.6%	197.8%	46.9%	63.5%	18.6%	15.3%
2019	34.6%	27.0%	89.8%	168.0%	57.5%	65.0%	19.2%	18.4%
2020	64.5%	42.9%	112.4%	262.9%	76.8%	111.3%	85.6%	2.2%

^*^Data here is from Baidu Index

**Fig. 2:**
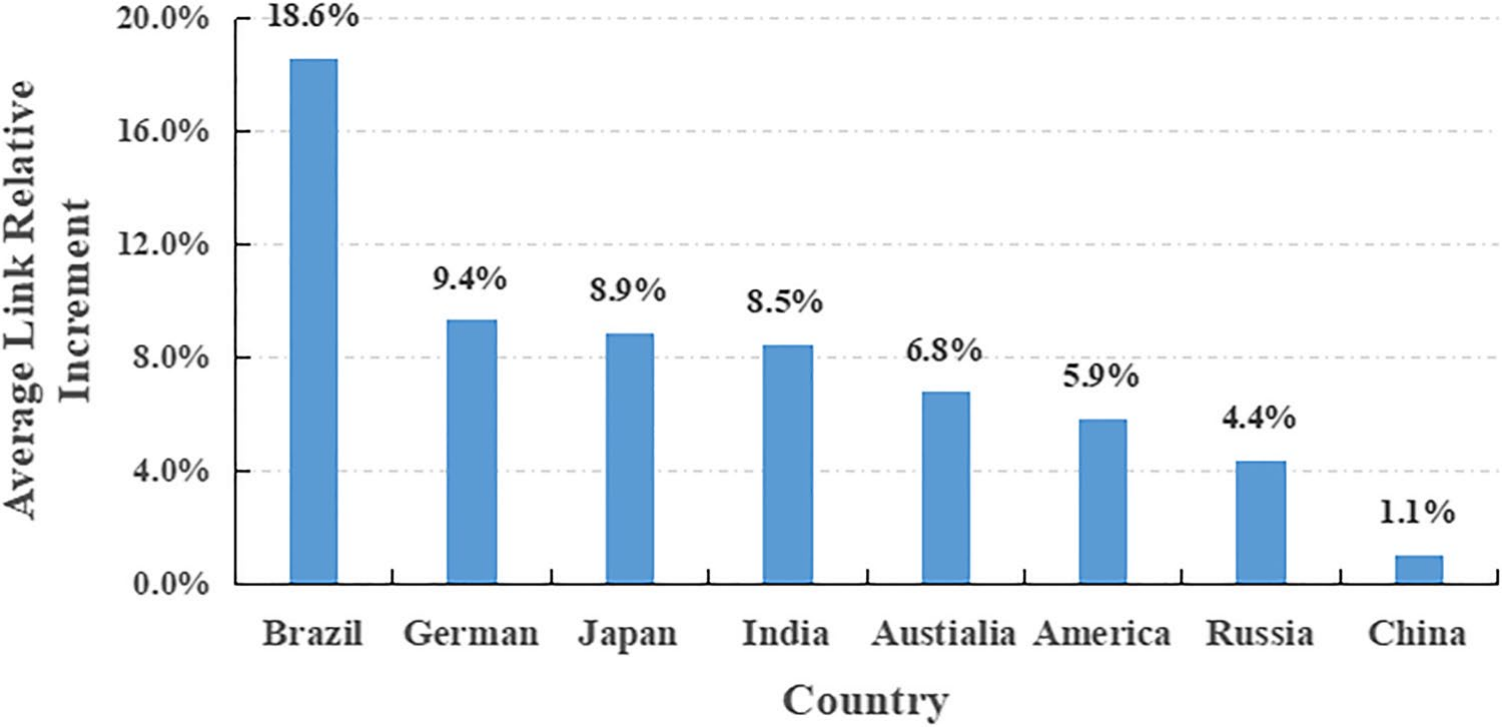
Average year-on-year SIS changes from 2011 to 2020

The results of the preliminary analysis of year-on-year changes in the SIS from 2011 to 2020 are presented in Table 2. Compared to the SIS in 2019, those for the USA, Russia, Japan, Brazil, Australia, Germany, India, and China increased by 22.3%, 12.4%, 11.9%, 35.4%, 12.3%, 28.0%, 55.8%, and − 13.7%, respectively, in 2020. The fastest years of SIS growth were 2020 (22.3%, USA), 2014 (14.5%, Russia), 2012 (19.0%, Japan), 2017 (93.5%, Brazil), 2014 (18.8%, Australia), 2015 (33.7%, Germany), 2020 (55.8%, India), and 2012 (36.3%, China), respectively. Notably, the SIS in India increased by 92.7% in 2017 (Table 2). Over the past decade, the SIS increased by an annual average of 18.6% (Brazil), 9.4% (German), 8.9% (Japan), 8.5% (India), 6.8% (Australia), 5.9% (USA), 4.4% (Russia), and 1.1% (China) (Fig. 2).

**Table 2:** Year-on-year changes in the search index for snoring (SIS) from 2011 to 2020

	America	Russia	Japan	Brazil	Australia	German	India	China
2011	-	-	-	-	-	-	-	-
2012	2.5%	12.2%	**19.0%**	− 2.2%	9.1%	11.7%	− 1.8%	**36.3%**
2013	0.4%	6.4%	11.2%	− 4.8%	− 3.2%	3.7%	11.0%	− 2.0%
2014	0.3%	**14.5%**	12.2%	4.2%	**18.8%**	2.3%	3.8%	− 9.5%
2015	13.2%	− 6.7%	8.8%	11.9%	15.1%	**33.7%**	18.5%	− 4.4%
2016	2.2%	− 10.8%	8.1%	32.5%	6.4%	− 8.1%	− 12.1%	3.5%
2017	11.8%	1.3%	3.6%	**93.5%**	1.2%	4.9%	3.0%	5.1%
2018	0.8%	0.0%	7.6%	7.0%	− 5.4%	7.1%	− 2.5%	− 8.3%
2019	0.0%	10.2%	− 2.5%	− 10.0%	7.2%	0.9%	0.5%	2.7%
2020	**22.3%**	12.4%	11.9%	35.4%	12.3%	28.0%	**55.8%**	− 13.7%

Bold values mean its maximum in this country

^*^ Data here is from Baidu Index

**Fig. 3:**
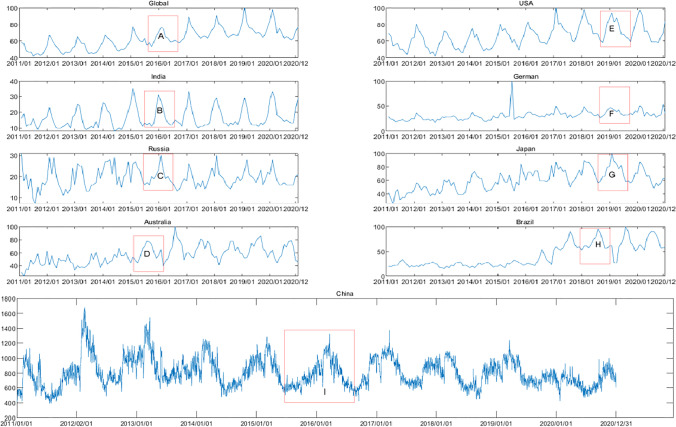
The variation in the search index for snoring (SIS) from 2011 to 2020 across several countries

**Fig. 4:**
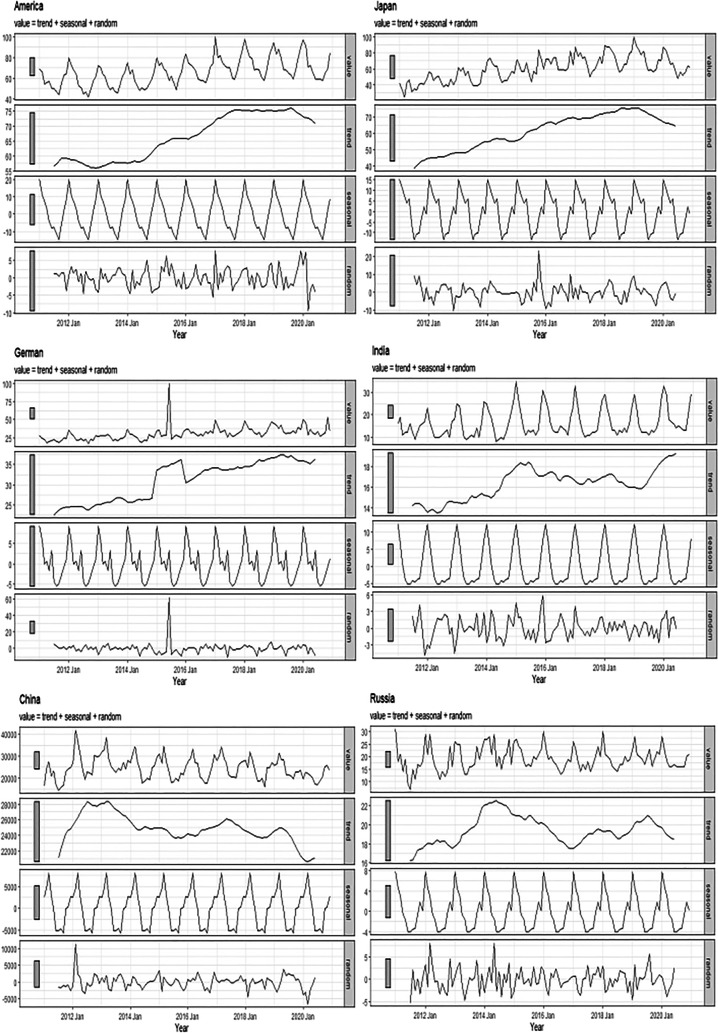
Seasonal decomposition for the search index for snoring (SIS) in America, Japan, German, India, China, and Russia

**Fig. 5:**
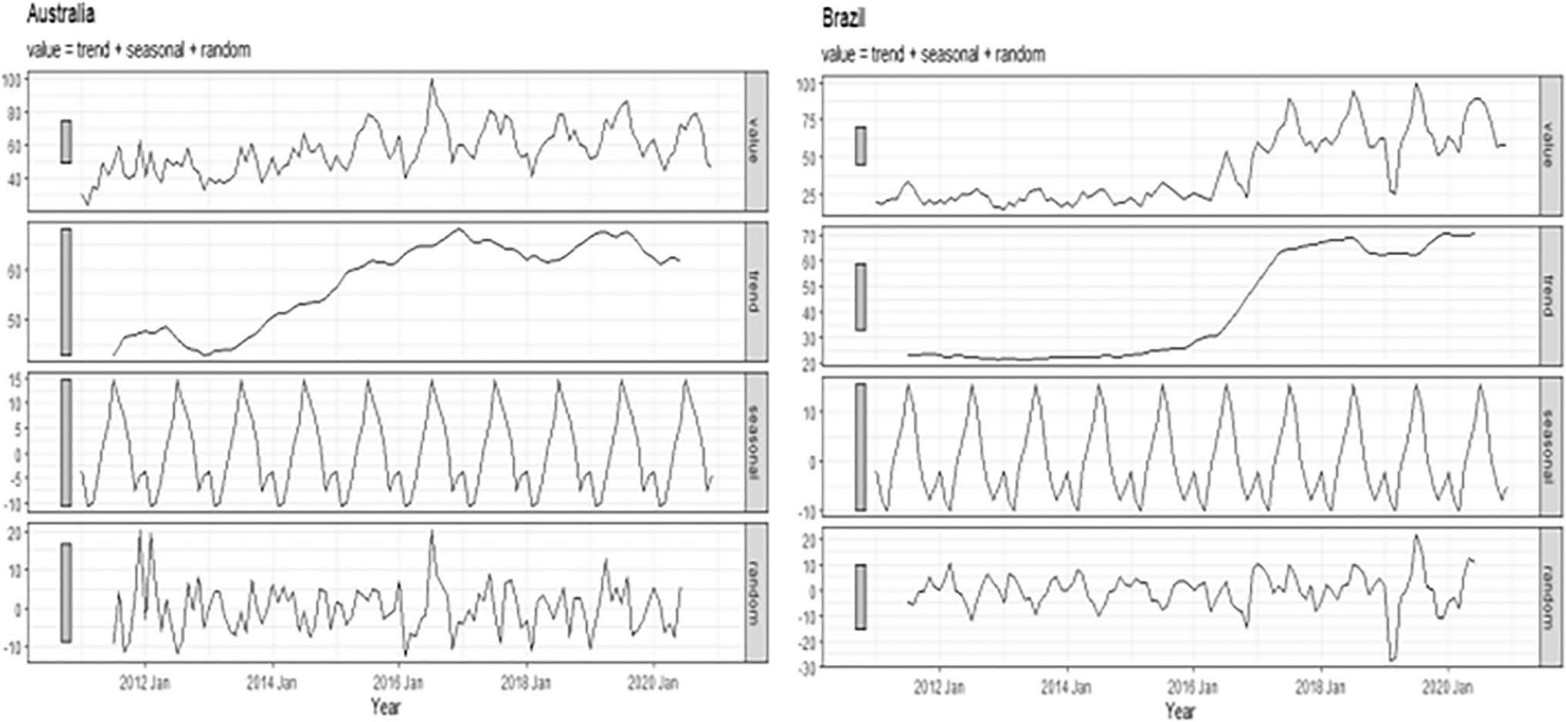
Seasonal decomposition for search index for snoring (SIS) in Australia and Brazil

Next, we evaluated monthly changes in the SIS and our analysis revealed that these SIS values changed cyclically (Fig. 3). Specifically, there was a clear peak in SIS global snoring levels (peak A). In countries in the northern hemisphere (USA, India, German, Russia, China, and Japan), SIS values increased from September to February and reduced from March to August (peaks B, C, E–G, H). On the other hand, in the southern hemisphere, they exhibited the opposite trend, with an increase from March to August and a decrease from September to February (peaks D, H). Time series decomposition analyses were performed for data from the northern and southern hemispheres (Figs. 4 and 5), indicating clear seasonal trends that differed among these countries. Notably, when the seasonal trends were not considered, an overall reduction in SIS values was evident since 2019 in the USA, Russia, Japan, and China (Fig. 4).

## Discussion

Snoring can result in complications such as fatigue, impaired concentration, daytime sleepiness, and decreased ability to work, with severe snoring often coinciding with the incidence of sleep apnea [4, 11, 34]. Reported rates of snoring prevalence range from 4.0 to 41.9%, although analyzing the trends in snoring incidence on a global scale is challenging [11, 15]. In the present study, internet search trend data were used to explore potential seasonal variations in the snoring search index, yielding some potentially interesting findings.

Many individuals obtain health-associated information from the internet, and the associated search data thus offers clear value to public health given that it is routinely updated and readily obtained [29, 30, 35]. This data can be used to construct estimates of health-related event distributions. For example, the use of internet-based data to monitor the spread of the COVID-19 pandemic has been widely reported [36, 37]. The Google Trends platform provides geospatial and temporal search volume pattern data that can be used for public health monitoring. While the Google Trends data are available for most countries globally, it is not accessible in China. On the other hand, the Baidu search engine is widely used in China, and it similarly collects data that can be used for epidemiological analysis. For example, Fang et al. explored the use of coronavirus epidemic-related keywords as a means of exploring real-time search data derived from the Baidu Index, concluding that the incorporation of these Baidu Index data was associated with significant improvements in the associated predictive model [30].

In the present study, “snoring” was used as a keyword to explore the association between seasonal changes and snoring, with the resultant data indicating that the SIS values in countries from both northern and southern hemispheres exhibited seasonal trends. Specifically, these SIS values were higher in winters or summers, during which the ambient air pollution levels increase. A previous study reported that the variation in concentrations of air pollutants such as PM_2.5_, PM_10_, and SO_2_ adopted a periodic U-shaped graph, where concentrations were higher in winters or summers [38]. This suggests a potential association between increased air pollution and snoring incidence. Consistent with this finding, another study that included 25,848 individuals in Sweden found that habitual snorers were more likely to report being bothered by air pollution compared to non-snorers (sometimes, 16.2% vs 13.9%; daily, 4.6% vs 3.1%), with similar results for traffic fumes (slightly more than 19% vs 18.5%; very, 5% vs 3.6%) [9]. Sánchez et al. also reported that higher ozone levels (OR: 1.693), humidity (OR: 1.161), and sulfur dioxide levels (OR: 1.16) were related to an increase in the risk of wheezing-related sleep disorders [28]. Prolonged indoor time spent in winters can also lead to increased exposure to secondhand smoke. A meta-analysis found that environmental tobacco smoke exposure was associated with an increased risk of habitual snoring (OR: 1.46, 95% CI, 1.29 to 1.65) [39]. Smoke can cause respiratory inflammation [40], potentially triggering or worsening breathing problems during sleep, thereby contributing to increased snoring incidence [41]. Smoking produces tar, nicotine, and carbon monoxide, which can irritate the pharyngeal mucosa and cause congestion or mucous membrane relaxation, thereby causing pharyngeal cavity constriction and eventually snoring. Leila et al. found higher habitual snoring frequencies among children residing in neighborhoods with the highest pollution levels showed an inverse relationship between ambient air quality and snoring in school-aged children [27]. Particulate matter (PM_2.5_ and PM_10_) and pollen can penetrate deep into human lungs, resulting in biological changes in the respiratory system, including the induction of inflammatory reactions, oxidative stress, and DNA damage, which could exacerbate the symptoms of respiratory diseases such as asthma, sleep apnea, and rhinitis [42, 43].

Lower temperatures may similarly contribute to such changes. A cross-sectional study reported lower temperature as a significant predictor of smoking more than 3 nights per week (OR = 0.865, 95% CI 0.751–0.997, *P* < 0.05) [28]. Moreover, a 10-year retrospective study reported a negative correlation between AHI and ambient temperature but it was directly correlated with atmospheric pressure, relative humidity, and carbon monoxide levels [44]. Furthermore, cold air stimulation is a common and significant factor in the acute exacerbation of chronic airway inflammatory disease [45]. Cold stimulation causes an acute exacerbation of the respiratory disease, resulting in a series of pathophysiological changes such as lung tissue infection, cell hypoxia, airway obstruction, inflammatory mediator infiltration, inflammatory factors chemotaxis, and excessive sputum secretion, as well as clinical manifestations such as severe cough, sputum coughing, and snoring. As reported in a long-term international study, COPD exacerbation increased almost twice in countries in the northern and southern hemispheres during the winter months [46]. It has been found that 66% of obstructive sleep apnea (OSA) occurs in patients with moderate to severe COPD [47] where snoring was the main clinical manifestation of OSA.

Winter is also a peak season for viral infections. Upper respiratory tract infection (URI) caused by viral infections could also lead to congestion and edema of the respiratory mucosa, thereby exacerbating upper airway obstruction and aggravating snoring [48]. Tiina M Mäkinen found that a 1 °C temperature drop increased the estimated risk of URI by 4.3%, the common cold by 2.1%, pharyngitis by 2.8%, and lower respiratory tract infection by 2.1%. A decrease in absolute humidity of 1 g/m^3^ increased the estimated risk of URI by 10.0% and pharyngitis by 10.8% [49]. The highest incidence of URIs was recorded during the cold season (December to March) throughout the study years. A study that included 15,413 people in Pakistan found that the winter season (December to March) had the highest incidence of respiratory tract infections between 2011 and 2016 [50]. Obesity might be another factor that contributes to snoring in winters. Winter weight gain is caused by a decrease in physical activity and an increase in caloric intake. Snoring can be caused by fat accumulation in the neck that narrows the windpipe.

**Fig. 6:**
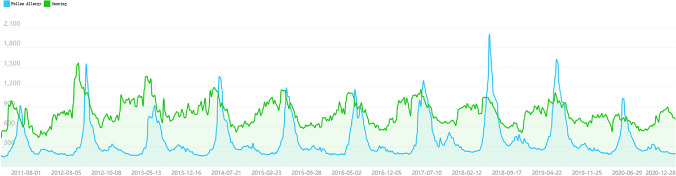
Baidu Index relevant to pollen allergy and snoring in mainland China from 2011 to 2020

As mentioned earlier, snoring as a clinical symptom can be caused by a variety of conditions, including pollen allergy. Pollen allergies usually occur in the spring and cause itchy eyes, runny nose, sneezing, blocked nose, and snoring. However, snoring caused by pollen allergy in spring has not been shown in Fig. 3. We speculated the following causes: (1) People may be aware that snoring is caused by allergies and may search for pollen allergy more than snoring during internet searches, which may lead to the absence of a peak in SIS searching for snoring in spring. (2) We are also studying the time series change in pollen allergy. For a better comparison between snoring and pollen allergy, see Fig. 6 in which snoring search and pollen allergy peaks almost overlap.

An interesting phenomenon of abrupt change in SIS in Brazil from 2016 to 2017–2018 was observed. The reasons might be that first, since 2016, Brazil’s economy started to recover and people’s income increased, boosting the popularity of smartphones and health concerns, leading to increased attention to snoring and search for more content about snoring. Second, Brazil has a large population of internet users. About 70% of Brazilians surf the internet and 90% of internet users go online every day. In addition, Brazil has the highest daily time spent on social media in the western hemisphere. Third, Brazilians love football and the 2016 World Cup was being held in Russia. Because of jet lag and daytime work, fans may have stayed up late after work to watch replays, leading to increased snoring.

The time series decomposition results showed that the SIS values decreased in the USA, China, Japan, Russia, and Australia in 2020. We speculated the following reasons: The overall increase in infections, complications, and fear of disease caused by COVID-19 in 2020 could have led to a decrease in attention to snoring. Measures such as home quarantine, temporary cabin quarantine, and hospitalization to prevent the spread of the pandemic may have led to unemployment, lower income, and lower living standards [51, 52], which might have forced people to pay more attention to the pandemic and reduce the online search for snoring. In particular, the SIS values in Germany changed insignificantly, and we speculated that this may be due to the low penetration of smartphones in Germany. According to a report in Statista, the number of smartphone users in Germany was 17.8 million in 2011, which accounts for 21.7% of the total German population [53].

## Conclusion

In this study, we determined the association between snoring and seasonal changes. Google Trends and Baidu indices were used to obtain a multi-country snoring search index from 2011 to 2020. We found that the snoring search index changed periodically, indicating that seasonal changes can affect snoring. The search index for snoring increased during cold season or the heating season. There are limitations to our study. First, our study was based on Internet trends and lacked data from the objective world to explain cold air, smoking, and other factors for each country. In our future study, we aim to collect real-world data to analyze the risk factors for snoring.

## Supplementary Information

Below is the link to the electronic supplementary material.

Supplementary file1 (RAR 6 KB)

## Data Availability

All data of this study is provided in the manuscript.
